# Probing the reaching–grasping network in humans through multivoxel pattern decoding

**DOI:** 10.1002/brb3.412

**Published:** 2015-10-21

**Authors:** Maria Grazia Di Bono, Chiara Begliomini, Umberto Castiello, Marco Zorzi

**Affiliations:** ^1^ Department of General PsychologyUniversity of PadovaPadovaItaly; ^2^ Cognitive Neuroscience CenterUniversity of PadovaPadovaItaly; ^3^ Centro Interdisciplinare Beniamino SegreAccademia dei LinceiRomaItaly; ^4^ IRCCS San Camillo HospitalVenice‐LidoItaly

**Keywords:** Functional magnetic resonance imaging, multivoxel pattern decoding, reaching‐only action, visuomotor reach‐to‐grasp action

## Abstract

**Introduction:**

The quest for a putative human homolog of the reaching–grasping network identified in monkeys has been the focus of many neuropsychological and neuroimaging studies in recent years. These studies have shown that the network underlying reaching‐only and reach‐to‐grasp movements includes the superior parieto‐occipital cortex (SPOC), the anterior part of the human intraparietal sulcus (hAIP), the ventral and the dorsal portion of the premotor cortex, and the primary motor cortex (M1). Recent evidence for a wider frontoparietal network coding for different aspects of reaching‐only and reach‐to‐grasp actions calls for a more fine‐grained assessment of the reaching–grasping network in humans by exploiting pattern decoding methods (multivoxel pattern analysis—MVPA).

**Methods:**

Here, we used MPVA on functional magnetic resonance imaging (fMRI) data to assess whether regions of the frontoparietal network discriminate between reaching‐only and reach‐to‐grasp actions, natural and constrained grasping, different grasp types, and object sizes. Participants were required to perform either reaching‐only movements or two reach‐to‐grasp types (precision or whole hand grasp) upon spherical objects of different sizes.

**Results:**

Multivoxel pattern analysis highlighted that, independently from the object size, all the selected regions of both hemispheres contribute in coding for grasp type, with the exception of SPOC and the right hAIP. Consistent with recent neurophysiological findings on monkeys, there was no evidence for a clear‐cut distinction between a dorsomedial and a dorsolateral pathway that would be specialized for reaching‐only and reach‐to‐grasp actions, respectively. Nevertheless, the comparison of decoding accuracy across brain areas highlighted their different contributions to reaching‐only and grasping actions.

**Conclusions:**

Altogether, our findings enrich the current knowledge regarding the functional role of key brain areas involved in the cortical control of reaching‐only and reach‐to‐grasp actions in humans, by revealing novel fine‐grained distinctions among action types within a wide frontoparietal network.

## Introduction

In the domain of motor control great attention has been given to reaching‐only and reach‐to‐grasp actions, apparently simple and straightforward behaviors which are part of our everyday life motor repertoire, and fundamental for our interaction with the environment.

A great extent of our knowledge regarding the cortical control of reach‐to‐grasp movements is rooted in neurophysiological studies on behaving monkeys, in which the activity of single neurons is recorded with techniques allowing a high level of spatial and temporal resolution. These studies have identified the main cortical structures involved in the control of visually guided reach‐to‐grasp movements. They are the primary motor cortex (F1), the premotor cortex (area F5), and the anterior part of the intraparietal sulcus (AIP; Murata et al. 1997, 2000). The ability to perform a successful reach‐to‐grasp action depends primarily on the integrity of F1; indeed, lesions of this area in macaques produce a remarkable deficit in the control of individual fingers, bringing to a loss of coordination abilities (Lawrence and Hopkins 1976). Area F5, which forms the rostral part of the macaque ventral premotor cortex (PMv) and AIP, a small zone lying within the rostral part of the posterior bank of the intraparietal sulcus (Matelli et al. 1985; Luppino et al. 1999; Matelli and Luppino 2001) are directly connected and are involved in converting intrinsic object properties (e.g., shape, size) into a proper hand conformation for grasping the object (Jeannerod et al., 1995).

In macaques trained to grasp various objects, activity of F5 and AIP neurons show not only strong similarities, but also important differences (Rizzolatti et al. 1988, 2002; Taira et al. 1990; Rizzolatti and Arbib 1998). On one side, both F5 and AIP neurons code for reach‐to‐grasp actions (Murata et al. 1997, 2000). However, AIP neurons seem to represent the entire action, whereas F5 neurons seem to be concerned with a particular segment of it (Rizzolatti et al. 1998; Murata et al. 2000). Another important difference is that visual responses to three‐dimensional objects are found more frequently in AIP than in F5 (Murata et al. 2000). This suggests that AIP, although part of a parieto‐frontal network dedicated to hand movements, also contains a population of neurons that code three‐dimensional objects in visual terms.

Building upon this knowledge, Fagg and Arbib (1998) suggest that AIP could store the objects' sensory properties (Taira et al. 1990; Murata et al. 1997, 2000). These representations influence the ventral premotor area F5 and also the dorsal premotor area F2, which is involved in visual guidance of the hand (Moll and Kuypers 1977; Godschalk et al. 1981; Weinrich and Wise 1982; Passingham 1987; Rizzolatti et al. 1988; Raos et al. 2004, 2006). Area F5 plays a primary role in selecting the most appropriate type of grip on the basis of the object affordances provided by AIP, thereby activating a motor representation of that object. This motor representation is then supplied to F2, which keeps memory of it and combines it with visual information provided by cortical areas of the superior parietal lobe to continuously update the configuration and orientation of the hand as it approaches the object. The final output is then sent to the F1 for motor execution (for review see Castiello and Begliomini 2008). Moreover, the same role of F2 is played by area V6A, which is strongly and reciprocally connected with the dorsal premotor cortex controlling arm movements, and elaborates visual information, motion and space, for controlling both reaching‐only and reach‐to‐grasp movements (Galletti et al. 2003; Fattori et al. 2009, 2010).

In humans, both functional magnetic resonance imaging (fMRI) and transcranial magnetic stimulation (TMS) studies have demonstrated the existence of localized cortical reach‐to‐grasp areas similar to those described in monkeys (Cavina‐Pratesi et al. 2007; Culham et al. 2006; Kroliczak et al. 2007; Tunik et al. 2007; for reviews see Castiello 2005; Castiello and Begliomini 2008; Filimon 2010). Overall, reach‐to‐grasp fMRI studies converge in considering the anterior part of the human intraparietal sulcus (hAIP), a likely homolog of monkey AIP (Grafton et al. 1996; Culham et al. 2003; Frey et al. 2005; Begliomini et al. 2007a; Hinkley et al. 2009). The key role of hAIP in the dynamic control of reach‐to‐grasp movements has also been confirmed in a series of TMS studies (Glover et al. 2005; Tunik et al. 2005; Rice et al. 2006). Tunik et al. (2005) have shown that applying TMS to the hAIP induces a delay in grasp adaptation, suggesting that this area performs a sort of iterative comparison between the incoming sensory information and the motor command during the ongoing movement.

The quest for the human homolog of macaque F5 has identified the ventral part of the premotor cortex (PMv) as a plausible candidate. However, neuroimaging studies investigating brain activity during a reach‐to‐grasp movement do not provide a coherent picture regarding the involvement of the PMv. Some fMRI studies have reported PMv activation during multidigit visually guided reach‐to‐grasp actions (Grol et al. 2007; Cavina‐Pratesi et al. 2010), object manipulation (Binkofski et al. 1999), and isometric grasping (Ehrsson et al. 2001), whereas other studies found no evidence of PMv involvement during visually guided reach‐to‐grasp action (Culham et al. 2006; Begliomini et al. 2007a,b). A possible explanation for this controversial finding, which contrasts with the clear involvement of PMv for reach‐to‐grasp movements in macaques (e.g., Rizzolatti et al. 1988), could be due to the fact that interspecies differences in the organization of the PMv, as well as the development of a motor speech area in humans, may have changed the location of the human functional homolog of monkey area F5 (Amunts and Zilles 2001). Moreover, it is worth noting that in the majority of studies, grasping‐related activity has been isolated by subtracting activations obtained during the reaching‐only from the reach‐to‐grasp task (Grafton et al. 1996; Culham et al. 2003; Frey et al. 2005; Begliomini et al. 2007a,b). Because in these studies both the reaching‐only and the reach‐to‐grasp tasks required specific motor goals—triggering premotor activity—it might well be that activations within premotor areas could have canceled one another when compared (Grafton et al. 1996; Culham et al. 2003; Frey et al. 2005; Begliomini et al. 2007a,b).

The dorsal part of the premotor cortex (PMd) has been suggested as the human correspondent of macaque area F2 (Matelli et al. 1991). As demonstrated in macaques (Raos et al. 2004), in humans the contribution of PMd to reach‐to‐grasp action is that of an online monitoring during the execution phase of the action. A study comparing reach‐to‐grasp movements with different levels of complexity, underlined bilateral PMd involvement in association with conditions that required higher levels of accuracy in implementing the action (Begliomini et al. 2007b).

Although the studies reviewed above significantly contributed to sketch an overall picture of the neural substrates of reaching‐only and reach‐to‐grasp in humans, a crucial issue that requires further investigation is how the different areas specifically contribute to the coding of grasp type (e.g., precision grasping [PG], whole hand grasping [WHG]) with respect to object size. This knowledge is fundamental in order to fully define the parallelism between the monkeys and the human grasping network. Indeed, Rizzolatti et al. (1988; see also Rizzolatti and Luppino 2001) showed that in monkeys, neurons within AIP and F5 areas code for grasping actions in relation to the type of object to be grasped. More in detail, F5 neurons seem to be mainly involved in selecting the most appropriate motor act from a “motor vocabulary.” For instance, the act of grasping a raisin (which requires the opposition of the index finger with the thumb) is encoded by neurons different from those that encode the grasping of an apple (which requires the opposition of the thumb with all fingers).

In humans, fMRI studies that directly contrasted PG versus WHG using conventional analysis, revealed activation differences between the two grasping actions in contralateral M1 (WGH > PG), bilateral PMv and hAIP (PG > WHG) (Ehrsson et al. 2000, 2001; Begliomini et al. 2007a). More recent studies have confirmed these findings, suggesting that grasp types (PG vs. WHG) have distinct representations within a wide frontal–parietal network subserving reach‐to‐grasp movements (Begliomini et al. 2014). This issue, however, remains controversial given that other studies failed to detect such differences (e.g., Kuhtz‐Buschbeck et al., 2008).

Another interesting question that requires further investigation is the role of object size in both reaching‐only and reach‐to‐grasp actions. The visuomotor channel hypothesis of Jeannerod (1981) states that the grasping action is composed of grip and transport components, which rely on intrinsic (e.g., object size) or extrinsic (e.g., location) object properties. According to this view, object size and location have to be processed independently in separate visual channels. However, the recent neuroimaging findings of Monaco et al. (2015) have suggested that, in humans, the cortical processing of object size and location does not conform to a strict segregation between grip and transport components of the reach‐to‐grasp action. In an fMRI adaptation paradigm, the authors found that left aIPS showed adaptation only to object size, whereas a wide frontoparietal network adapted to both object size and location. Furthermore, in an electroencephalogram (EEG)/event‐related potentials (ERP) study, Tarantino et al. (2014) showed that the kinematics of reaching‐only, as well as the amplitude and the latency of P300 and N400 ERP components in parietal and prefrontal sites, respectively, were modulated by object size, consistent with physiological findings on nonhuman primates (Fattori et al. 2012). The possibility to shed further light on these issues is offered by a multivariate approach that exploits multivoxel pattern analysis (MVPA; e.g., Di Bono and Zorzi 2008; O'Toole et al. 2007; Pereira et al. 2009; Zorzi et al. 2011). A study by Gallivan et al. (2011) showed distinct activity patterns coding different precision grasping actions toward two differently sized objects positioned at two different spatial locations (i.e., the smaller cube on the top of the larger one). The authors claimed that it was possible to decode two different types of grasping, but it was unclear whether this result could be related to the object size or to a different direction in reaching‐only toward the bottom or top object. Gallivan et al. (2011), also showed that voxel pattern activity within multiple frontoparietal areas during movement planning allowed discrimination between reach‐to‐grasp and reaching‐only actions. More evidence against a clear distinction between a dorsomedial (e.g., superior parieto‐occipital cortex [SPOC], medial intraparietal area MIP, and PMd) and a dorsolateral (e.g., hAIP and PMv) pathway, specialized for reaching‐only and grasping, respectively, was provided by Fabbri et al. (2014). These recent findings in humans are consistent with the theory of a dorsomedial visual stream (e.g., V6A) involved in reach‐to‐grasp actions, suggested by Galletti et al. (2003). Indeed, this has been documented by Fattori et al. (2009) and more directly by Fattori et al. (2010), who showed evidence of grasping neurons in the medial parieto‐occipital cortex of the macaque monkeys. The abovementioned results about macaque area V6A suggested SPOC area as its putative homolog in humans (Pitzalis et al. 2013, 2015; Tosoni et al. 2014). The human homolog of V6A has been also identified as the parieto‐occipital junction by Prado et al. (2005) and as the superior end of the parieto‐occipital sulcus (sPOS) by Filimon et al. (2009).

The recent findings on different aspects of reaching‐only and reach‐to‐grasp actions call for a thorough and fine‐grained assessment of the reaching–grasping network in humans. We exploited pattern decoding methods for investigating the following key questions: (1) whether there are distinct representations for different grasp types (i.e., PG vs. WHG); (2) whether there are distinct representations of object size during reaching‐only action; (3) whether object size could modulate each grasp type action in a congruent/incongruent action setting (e.g., PG toward a small object and WHG toward a large object [congruent] vs. PG toward a large object and WHG toward a small object [incongruent]). Moreover, we aimed at: (4) replicating the findings of Gallivan et al. (2011) and Fabbri et al. (2014), which provided evidence of distinct representations for reaching‐only and reach‐to‐grasp actions, distributed across a wide frontoparietal network; (5) replicating the findings of Monaco et al. (2015) on the representation of the object size during reach‐to‐grasp actions.

To address these issues, we reanalyzed the fMRI data of Begliomini et al. (2007b) using MVPA for investigating the specific contribution of each brain area belonging to the reaching–grasping network in humans. To this end, we selected anatomically defined regions of interest (ROIs) within a wide frontoparietal network involved in reaching‐only and reach‐to‐grasp action representation (e.g., Gallivan et al. 2011; Fabbri et al. 2014). We then trained a support vector machine (SVM) classifier (see Pereira et al. 2009, for a tutorial overview) with linear kernel on the voxel pattern activity of those ROIs for decoding (1) object size in both reach‐to‐grasp and reaching‐only actions, (2) grasp type, (3) the congruence between grasp type and object size, and (4) the action type (i.e., reach‐to‐grasp vs. reaching‐only actions).

## Materials and Methods

### Participants

Nineteen right‐handed participants (12 female; 19–30 years old) participated in the experiment. All gave written informed consent before entering in the scanner room. According to Begliomini et al. (2007b), three participants were not included in the analysis due to the presence of head motion. The cut‐off used for motion correction tolerance was the size of the voxel (3.3 × 3.3 × 3 mm). In other words, if motion exceeded these measures in translation and/or rotation, the participant was not included in the analysis. All participants were right‐handed as measured by the Edinburgh Handedness Inventory (Oldfield 1971). The experimental procedures were approved by the ethics committee of the University of Padua (see Begliomini et al. 2007b, for all details).

### Apparatus

Participants were requested to perform either reaching‐only or reach‐to‐grasp actions toward stimuli presented by using a metal‐free apparatus, which was composed of a table mounted on a plexiglass structure that allowed the presentation of real 3D stimuli to participants lying supine in the scanner. Participants had their head tilted at an angle of ~30° and they were supported by a foam wedge permitting direct viewing of the stimulus without mirrors. The apparatus was placed at a natural reaching distance (~15 cm) above the participant's pelvis for avoiding further movements of the upper part of the trunk.

### Stimuli and task procedures

The stimuli consisted of two spherical plastic objects of different dimensions (small stimulus: 3 cm diameter; large stimulus: 6 cm diameter). Participants were requested to perform three different actions toward either the small or the large stimulus: (1) grasping the stimulus with a PG; (2) grasping the stimulus with a WHG; (3) only reach the stimulus (R), by touching it with the hand knuckles, maintaining the hand closed like in a fist. Participants were informed about the type of movement to perform through a sound delivered by pneumatic MR‐compatible headphones: (1) PG—low tone (duration: 200 msec; frequency: 1.7 kHz); (2) WHG—high tone (duration: 200 msec; frequency: 210 Hz); R‐double tone (duration: 70 msec each, staggered by a 60 msec silence period; frequency: 445 Hz) and they were instructed to start their action toward the stimulus only when the sound was delivered.

### Experimental design

**Figure 1:**
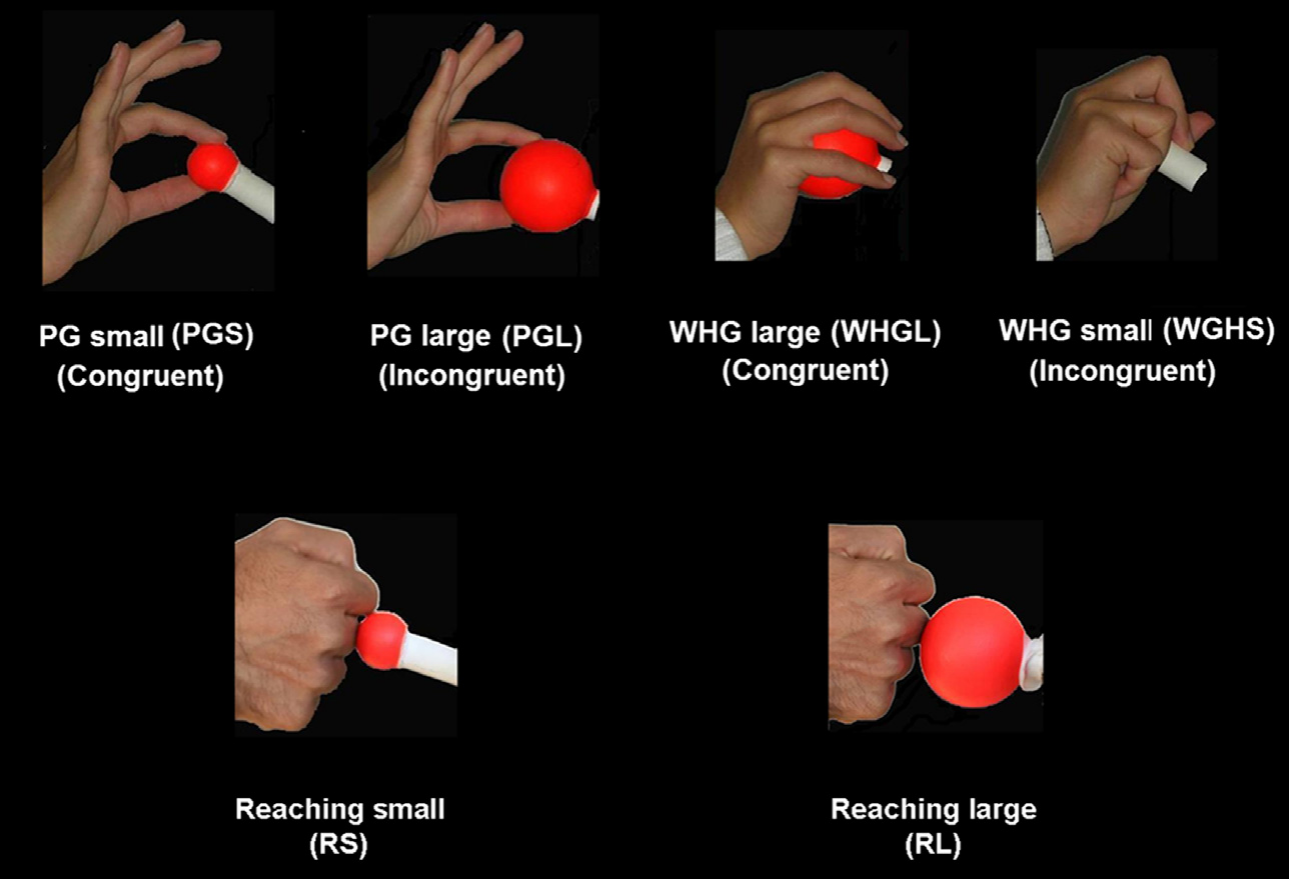
Experimental conditions (adapted from Begliomini et al. 2007b). Participants viewed one of the two stimuli (i.e., a spherical object of two different sizes) and performed three different tasks (i.e., reaching‐only and two types of reach‐to‐grasp actions). The experimental conditions involved either precision grasp (PG), whole hand grasp (WHG), or Reaching‐only (R) actions. Participants were instructed about the movement to perform (PG, WHG, and R) with a sound delivered through headphones. According to the size of the object to be grasped, the reach‐to‐grasp action was defined as congruent (PG toward a small object—PGS; WHG toward a large object—WHGL) or incongruent (PG toward a large object—PGL; WHG toward a small object—WHGS). All actions had to be performed with the right hand.

The experiment was conducted by using an event‐related design. inter stimulus interval (ISI) varied from 3 to 8 sec with a “long exponential” probability distribution (Hagberg et al. 2001). ISIs distribution was fully randomized across trials in each run for each subject. Action toward the stimulus (PG, WHG, R) and stimulus dimension (small or large) were manipulated as to create six different conditions (see Fig. 1): (1) “PG toward the small object” (PGS); (2) “PG toward a large object” (PGL); (3) “WHG toward a large object” (WHGL); (4) “WHG toward a small object” (WHGS); (5) “reaching‐only toward a small object” (RS); (6) “reaching‐only toward a large object” (RL). There were 45 trials for each experimental condition, grouped into mini‐blocks of five trials belonging to the same condition. Trials were divided in four runs, with a short rest between each run. In the odd runs the object was small, whereas in the even runs the object was large.

### Imaging parameters

Images were acquired with a whole‐body 3T scanner (Siemens Magnetom Trio, TIM system, Siemens, Erlangen, Germany) equipped with a standard Siemens 12 channels coil. Functional images were acquired with a gradient‐echo, echo‐planar (EPI) T2*‐weighted sequence in order to measure blood oxygenation level‐dependent (BOLD) contrast throughout the whole brain (47 contiguous axial slices acquired with descending interleaved sequence, 64 × 64 voxels, 3.3 × 3.3 × 3 mm resolution, FOV = 210 × 210 mm, flip angle = 90°, TE = 30 msec). Volumes were acquired continuously with a repetition time (TR) of 3 sec; 117 volumes were collected in each single scanning run (5:51 min; four scanning runs in total). High‐resolution T1‐weighted images were acquired for each subject (3D MP‐RAGE, 176 axial slices, data matrix 256 × 256, 1 mm isotropic voxels, TR = 1859 msec, TE = 3.14 msec, flip angle = 22°).

### Regions of interest

The functional images were preprocessed using the software package SPM (Wellcome Department of Imaging Neuroscience, University College of London, http://www.fil.ion.ucl.ac.uk/spm/). For each participant, images underwent motion correction and unwarping, and each volume was realigned to the first volume in the series. The mean of all functional images was then co‐registered to the anatomical scan, previously corrected for intensity inhomogeneity. EPI images were then normalized adopting the MNI152 template, supplied by the Montreal Neurological Institute (http://www.mni.mcgill.ca/) and distributed with the software SPM. To avoid any circularity issue in ROI selection (Kriegeskorte et al. 2009), we did not rely on the functional data but selected six ROIs that were defined on purely anatomical grounds (using the SPM Anatomy toolbox; http://www.fil.ion.ucl.ac.uk/spm/ext/#Anatomy). One additional ROI, selected on the basis of the results of Fabbri et al. (2012), was obtained through a spherical image mask using the SPM SimpleROIBuilder toolbox (http://www.fil.ion.ucl.ac.uk/spm/ext/#SimpleROIBuilder).

The seven ROIs were defined as follows:

- ROI‐1: bilateral superior parieto‐occipital cortex (SPOC) defined according to the functional study by Fabbri et al. (2012). We extracted a sphere of 8‐mm radius, centered on the Talairach coordinates (SPOC LH: −17, −72, 37; SPOC RH: 21, −73, 31).
- ROI‐2: bilateral superior parietal lobe (SPLap), defined according to the anatomical study by Scheperjans et al. (2008). We used two different subregions of SPL (labeled as SPL 7A and SPL 7P in the Anatomy toolbox) to create this anatomical mask.
- ROI‐3: bilateral hAIP, defined according to the anatomical study by Choi et al. (2006) on the human IPS. We used three different subregions of the anterior IPS (labeled as hIP1, hIP2, and hIP3 in the Anatomy toolbox) to create this anatomical mask.
- ROI‐4: bilateral Brodmann area (BA) 1/2/3ab, according to the anatomical studies of Geyer et al. (1999, 2000) and Grefkes et al. (2001).
- ROI‐5: bilateral primary motor cortex, defined according to Geyer et al. (1996), but selecting only the posterior part of the primary motor cortex (bilateral BA 4p) to focus on the hand representation.
- ROI‐6: bilateral premotor area BA 6, defined according to the anatomical study of Geyer (2003), roughly corresponding to the PMd. Specifically, BA 6 is a rather large area that includes not only PMd laterally, but also supplementary motor area (SMA) and pre‐SMA medially.
- ROI‐7: bilateral BA 44/45, according to Amunts et al. (1999), roughly corresponding to the PMv.

To test classifier performance outside our selected network, we defined one additional control ROI in which no BOLD signal was expected and then no consistent classification performance should be possible (see Gallivan et al. 2011, for a similar methodological procedure). Therefore, we selected a (8 mm)^3^ cubic region outside the skull of the brain (centroid MNI coordinates: [63, 63, 75]).

### Preprocessing

After ROI extraction, the voxel time series were preprocessed through a series of commonly used steps: standardization, detrending, and temporal filtering. For each participant, each of the four runs was processed separately. The time series were first standardized in order to have zero mean and standard deviation 1. Then, linear trends in each time series were removed, and a high‐pass filter (0.01 Hz) was applied in order to remove low frequency drift in the signal.

### Classifier analysis

We used SVM with linear kernel (the C parameter was fixed to 1, which is the default value) as multivoxel pattern classifier. We performed six classifications: (1) *Object size in Reach‐to‐grasp* (i.e., PGS + WHGS vs. PGL + WHGL); (2) *Object size in Reaching‐only* (i.e., RS vs. RL); (3) *Grasp type* (i.e., PGS + PGL vs. WHGS + WHGL); (4) *Congruence* between grasp type and object size (i.e., PGS + WHGL [Congruent] vs. PGL + WHGS [Incongruent]); (5) *PG vs. Reaching‐only* (i.e., PGS + PGL vs. RS + RL); (6) *WHG* vs. *Reaching‐only* (i.e., WHGS + WHGL vs. RS + RL). For each participant, we trained a linear classifier on the voxels within each selected ROI, separately for each hemisphere. We used only the fMRI volumes corresponding to the experimental conditions for each classification (e.g., grasp type: PG vs. WHG) as input to the classifier. In order to maintain sample independence for SVM training and testing, for each mini‐block (i.e., five trials from the same condition), we discarded the first four volumes to capture a stable fMRI signal without incorporating any noise from trials within the previous mini‐block and then created one sample averaging the remaining volume images (e.g., Pereira et al. 2009). Consequently, the target condition, relative to each contrast, was coded in a way to have a vector ***T***_*i*_ {+1, −1}_*i* = 1,…,*N*_, where *i* refers to the sample and *N* is the number of samples relative to both conditions in the classification (e.g., *N* = 36 in PG vs. WHG classification), in which all the samples corresponding to one target condition (e.g., PG) were labeled with +1, whereas all the other samples (e.g., WHG) with −1. Cross‐validation was used to estimate the test generalization performance. The SVM classifier was trained on the data set using a modified version of leave‐one‐out cross‐validation. At each step of the cross‐validation loop, two samples (one for each condition) were excluded from the training set and used to test generalization performance (see Zorzi et al. 2011). Classifier accuracy, computed across the entire cross‐validation loop on the test set, was used as statistical measures of binary classification.

### Statistical analysis on the classifier performance

Previous studies (e.g., Chen et al. 2011; Gallivan et al. 2011) showed that *t*‐test group analysis, with respect to nonparametric randomization tests, is a rather conservative estimate of significant decoding accuracy. Therefore, we conducted a set of one‐tailed *t*‐tests, one for each ROI, on the classifier accuracy (against the chance level of 50%) to obtain group statistics regarding the discrimination between the two conditions included in each classification. We used false discovery rate (FDR) for correcting for multiple comparisons. Furthermore, for each classification we assessed the possible differences between ROIs and hemispheric asymmetries by performing an ANOVA on the classifier accuracy using ROI (SPOC, SPLap, hAIP, BA 1/2/3ab, BA 44/45, BA 6, BA 4p) and hemisphere (left vs. right) as factors. Finally, to assess the sensitivity of each ROI for each classification, we performed a repeated measure (RM) ANOVA on the classifier accuracy, using classification as a within‐subject factor.

## Results

In this section we report, for each classification (i.e., Object size in reach‐to‐grasp action, Object size in reaching‐only action, Grasp Type, Congruence, PG vs. Reaching‐only, WHG vs. Reaching‐only) the results obtained by training linear SVM classifiers on each selected ROI, separately for the left and the right hemisphere. For each ROI, the results are expressed in terms of classification performance on the test set.

### Object size in reach‐to‐grasp action

Independently from the grasp type, it was not possible to discriminate between grasping a small and large object from all the selected ROIs in both hemispheres, Control ROI included (mean accuracy = 0.47 ± 0.02 SEM, all *t*s < 0.59).

### Object size in reaching‐only action

It was not possible to discriminate between reaching‐only a small and large object from all the left and right selected ROIs, Control ROI included (mean accuracy = 0.53 ± 0.03 SEM, all *t*s < 2.3).

### Grasp type

Results for grasp type classification are summarized in Table 1.

**Table 1:** Grasp type classification. Results obtained by training linear SVM classifiers on each selected ROI, separately for the left and the right hemisphere. For each ROI, the results are expressed in terms of classification performance on the test set (M ± 1 SEM) and the *t* statistics for assessing classification significance

ROI	Left hemisphere	Right hemisphere
SPOC	.52 ± .03 *t*(15) = 0.75, ns	.55 ± .03 *t*(15) = 1.49, ns
SPLap	.61 ± .02 *t*(15) = 4.55, *P* < .001	.54 ± .03 *t*(15) = 3.55, *P* < .01
hAIP	.57 ± .03 *t*(15) = 2.32, *P* = .017	.51 ± .02 *t*(15) = .48, ns
BA 1/2/3ab	.67 ± .02 *t*(15) = 7.31, *P* < .0001	.59 ± .02 *t*(15) = 3.92, *P* < .0001
BA 4p	.56 ± .02 *t *=* *2.4, *P *=* *.015	.56 ± .02 *t*(15) = 2.54, *P *=* *.015
BA 6	.6 ± .2 *t*(15) = 2.95, *P* < .001	.56 ± .03 *t*(15) = 2.14, *P *=* *.025
BA 44/45	.58 ± .03 *t*(15) = 2.99, *P* < .005	.57 ± .03 *t*(15) = 2.42, *P = *.015
Control ROI	.5 ± .02, *t *= −.17, ns	

SVM, support vector machine; ROI, regions of interest; SPOC, superior parieto‐occipital cortex; SPLap, superior parietal lobe; BA, Brodmann area; hAIP, anterior part of the human intraparietal sulcus.

**Figure 2:**
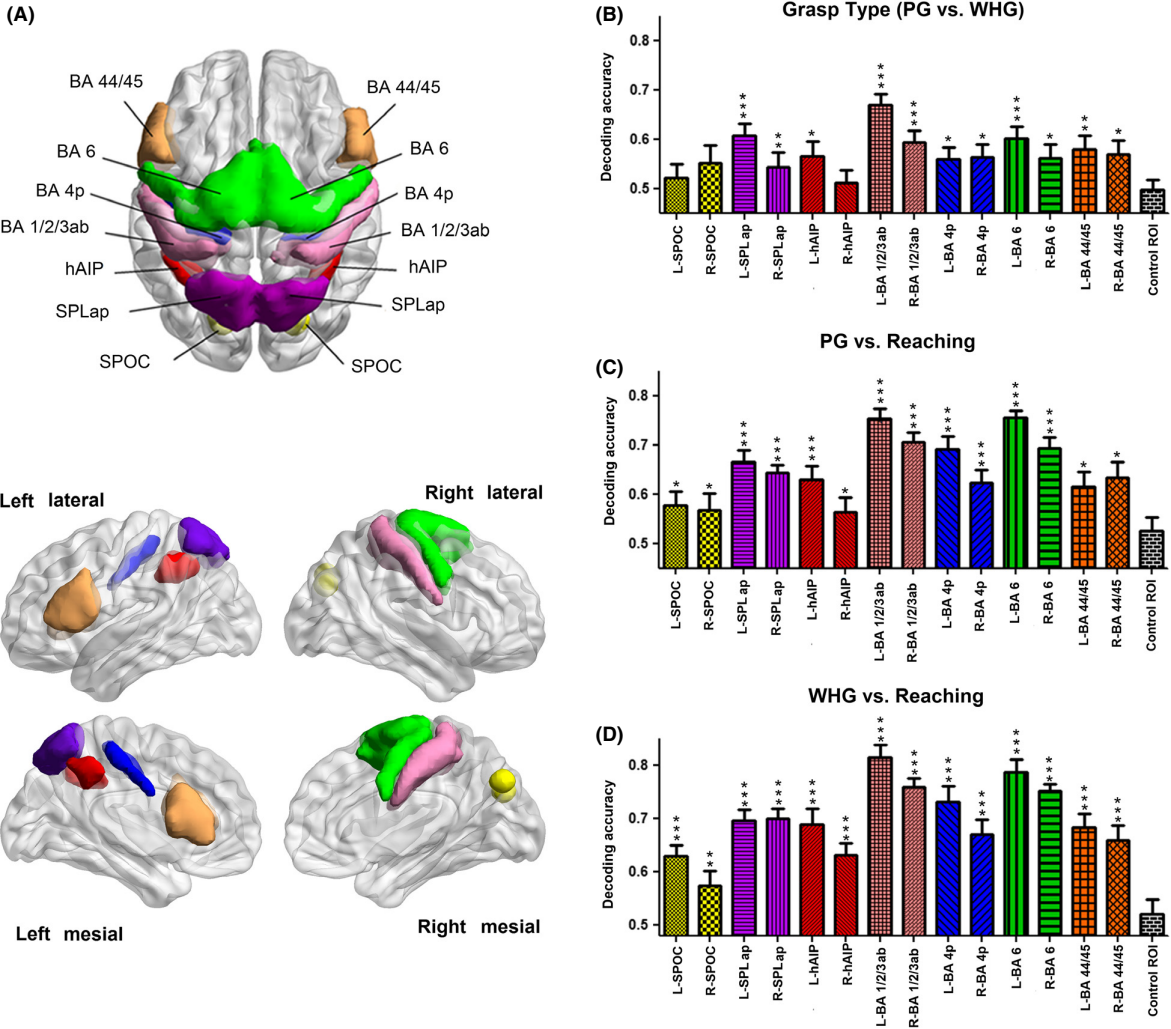
(A) Regions of interest (ROIs) used in the multivariate classifier analyses, transparently superimposed on top, lateral and mesial view of a standard template using BrainNet Viewer (http://www.nitrc.org/projects/bnv/) (Xia et al. 2013). ROI‐1 (yellow) includes SPOC areas (Fabbri et al. 2012). ROI‐2 (violet) includes SPLap areas (Scheperjans et al. 2008). ROI‐3 (red) includes three subregions in the hAIP (Choi et al. 2006). ROI‐4 (pink) includes BA 1/2/3ab (Geyer et al. 1999, 2000; Grefkes et al. 2001). ROI‐5 (blue) includes the posterior part of the BA 4 (Geyer et al. 1996). ROI‐6 (green) includes BA 6 (Geyer 2003). ROI‐7 (orange) includes BA 44/45 (Amunts et al. 1999). (B) Mean linear SVM classification accuracy for grasp type decoding as a function of the involved ROIs in the left (L) and right (R) hemisphere. (C) Mean linear SVM classification performance for discriminating (independently from the object size) between PG and Reaching‐only conditions as a function of the involved ROIs in each hemisphere. (D) Mean linear SVM classification performance for discriminating (independently from the object size) between WHG and Reaching‐only conditions as a function of the involved ROIs in each hemisphere. Error bars indicate one standard error of the mean. Asterisks assess statistical significance with one‐tailed t tests across subjects with respect to 50% (significance levels: *P < .05; **P < .01; ***P < .001 ).

The classifier analyses showed that it was possible to linearly decode the type of grasp from the voxel pattern activity of all the selected ROIs with the exception of bilateral SPOC, right hAIP, and the control ROI (see Table 1; Fig. 2, panel B), revealing an hemispheric asymmetry for the hAIP.

**Figure 3:**
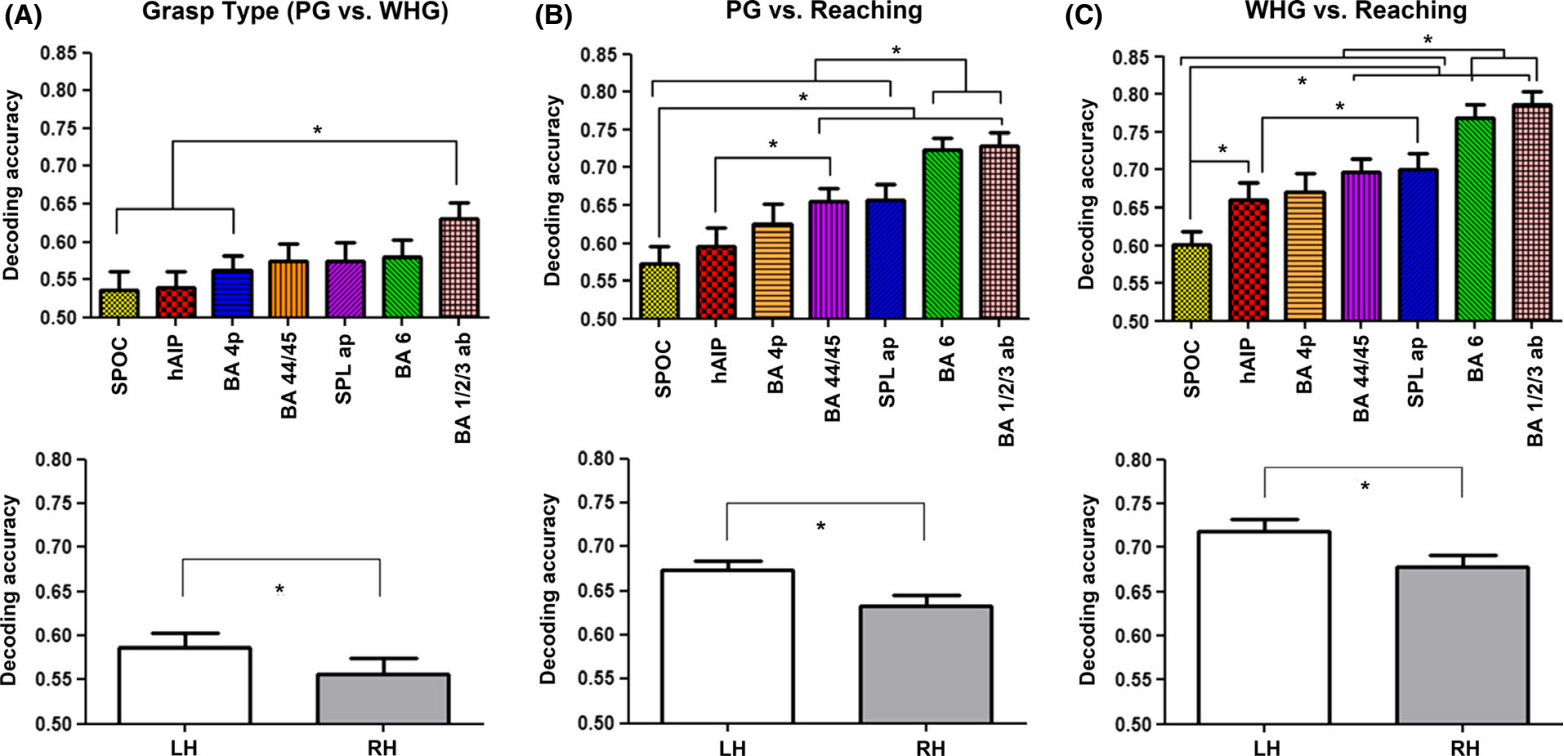
Results of the RM‐ANOVA on the decoding accuracy. (A) Grasp type: independently from the hemisphere, decoding from somatosensory areas was significantly more accurate than from SPOC, hAIP, and BA 4p. (B) PG versus Reaching‐only: independently from the hemisphere, decoding from somatosensory areas and BA 6 was significantly more accurate than from SPOC and hAIP. Moreover, decoding accuracy from voxel pattern activity of BA 6 was significantly higher than from SPLap. (C) WHG versus Reaching‐only: independently from the hemisphere, decoding from somatosensory areas was significantly more accurate than from all the other ROIs. In contrast, decoding accuracy from SPOC areas was significantly lower than that from all the other ROIs. Moreover, decoding from BA 6 was significantly more accurate than from hAIP and BA 44/45. For all the three classifications, independently from the selected ROI, the decoding accuracy was significantly higher in the left (contralateral) hemisphere than in the right (ipsilateral) hemisphere (see the bottom part of each panel). Error bars indicate one standard error of the mean across subjects. Asterisks assess statistical significance levels, as reported in the Result section.

To investigate possible interhemispheric asymmetries for the ROIs, we performed RM‐ANOVA on the classifier accuracy using ROI (SPOC, SPLap, hAIP, BA 1/2/3ab, BA 44/45, BA 4p, and BA 6) and hemisphere (left vs. right) as within‐subject factors. The analysis revealed a main effect of ROI (*F*(6, 90) = 4.03, *P* = 0.001, $\eta_{p}^{2}$ = 0.21) and hemisphere (*F*(1, 15) = 8.12, *P* = 0.012, $\eta_{p}^{2}$ = 0.35). The two‐way interaction was not significant (*F* = 1.49). Decoding accuracy was higher when decoding from the left (*M* = 0.59 ± 0.02 SEM) than from the right (*M* = 0.57 ± 0.02 SEM) hemisphere. Paired *t*‐tests (FDR corrected, corrected *α* = 0.007) showed higher decoding accuracy in the somatosensory cortex (BA 1/2/3 ab) (*M* = 0.63 ± 0.02 SEM) with respect to SPOC (*M* = 0.54 ± 0.02 SEM, *t*(15) = 3.73, *P *=* *0.002), hAIP (*M* = 0.54 ± 0.02 SEM, *t*(15) = 4.78, *P* < 0.001), and the selected motor areas (BA 4p) (*M* = 0.56 ± 0.02 SEM, *t*(15) = 4.2, *P *=* *0.001) (see Fig. 3, panel A). No further significant results were observed.

### Congruence

From none of the left and right selected ROIs (Control ROI included), it was possible to discriminate between congruent and incongruent conditions (mean accuracy = 0.48 ± 0.02 SEM, all *t*s < 0.78).

### Precision grasping versus reaching

Results for reach‐to‐grasp using PG versus reaching‐only classification are summarized in Table 2.

**Table 2:** PG versus reaching classification. Results obtained by training linear SVM classifiers on each selected ROI, separately for the left and the right hemisphere. For each ROI, the results are expressed in terms of classification performance on the test set (M ± 1 SEM) and the *t* statistics for assessing classification significance

ROI	Left hemisphere	Right hemisphere
SPOC	.58 ± .03 *t*(15) = 2.72, *P *=* *.016	.57 ± .03 *t*(15) = 1.96, *P *=* *.034
SPLap	.67 ± .03 *t*(15) = 6.65, *P *<* *.001	.64 ± .02 *t*(15) = 9.34, *P *<* *.001
hAIP	.63 ± .03 *t*(15) = 4.41, *P *=* *.001	.56 ± .02 *t*(15) = 2.26, *P* = .039
BA 1/2/3ab	.75 ± .02 *t*(15) = 12.9, *P *<* *.0001	.71 ± .02 *t*(15) = 1.27, *P *<* *.0001
BA 4p	.69 ± .03 *t*(15) = 7.39, *P *<* *.0001	.62 ± .03 *t*(15) = 4.68, *P *<* *.0001
BA 6	.69 ± .02 *t*(15) = 8.22, *P *<* *.0001	.62 ± .03 *t*(15) = 4.68, *P *=* *.019
BA 44/45	.62 ± .03 *t*(15) = 4.68, *P *=* *.019	.63 ± .03 *t*(15) = 4.02, *P *<* *.0001
Control ROI	.52 ± .03, *t *=* *.85, ns	

SVM, support vector machine; PG, precision grasping; ROI, regions of interest; SPOC, superior parieto‐occipital cortex; SPLap, superior parietal lobe; BA, Brodmann area; hAIP, anterior part of the human intraparietal sulcus.

The classifier analyses showed that, independently from the object size, it was possible to linearly discriminate between PG and Reaching‐only from the voxel pattern activity of all the selected ROIs with the exception of the control ROI (see Table 2; Fig. 2, panel C).

To investigate possible interhemispheric asymmetries, we performed an RM‐ANOVA on the classifier accuracy using ROI (SPOC, SPLap, hAIP, BA 1/2/3ab, BA 44/45, BA 4p, and BA 6) and hemisphere (left vs. right) as within‐subject factors. The analysis revealed a main effect of ROI (*F*(6, 90) = 9.38, *P* = 0.001, $\eta_{p}^{2}$ = 0.39) and hemisphere (*F*(1, 15) = 7.71, *P* = 0.014, $\eta_{p}^{2}$ = 0.34). The two‐way interaction was not significant (*F* = 1.31). Independently from the selected ROI, classifier accuracy was higher when decoding from the left (*M* = 0.67 ± 0.01) than from the right (*M* = 0.63 ± 0.01) hemisphere. Paired *t*‐tests (FDR corrected, corrected *α* = 0.031) showed that decoding accuracy from BA 1/2/3ap (*M* = 0.73 ± 0.02 SEM) and BA 6 (*M* = 0.72 ± 0.02 SEM) was higher with respect to all the other ROIs (all *t*s* *≥ 4.5, all *P*s < 0.015). Furthermore, higher accuracy was observed when decoding from SPLap (*M* = 0.65 ± 0.02 SEM) with respect to hAIP (*M* = 0.6 ± 0.02 SEM, *t*(15) = 5.63, *P* < 0.0001). Finally, decoding accuracy from SPOC areas was lower than those obtained from all of the other ROIs (all *t*s ≤ 2.8) with the exception of hAIP (*t* = 0.84) and BA 44/45 (*M* = 0.63 ± 0.03 SEM, *t *=* *1.6) (see Fig. 3, panel B). No further significant results were observed.

### Whole hand grasping versus reaching

Results for reach‐to‐grasp using WHG versus Reaching‐only classification are summarized in Table 3.

**Table 3:** WHG versus reaching classification. Results obtained by training linear SVM classifiers on each selected ROI, separately for the left and the right hemisphere. For each ROI, the results are expressed in terms of classification performance on the test set (M ± 1 SEM) and the *t* statistics for assessing classification significance

ROI	Left hemisphere	Right hemisphere
SPOC	.63 ± .02 *t*(15) = 5.99, *P *<* *.001	.57 ± .03 *t*(15) = 2.64, *P *<* *.005
SPLap	.7 ± .02 *t*(15) = 1.32, *P *<* *.001	.7 ± .02 *t*(15) = 13.47, *P *<* *.001
hAIP	.69 ± .03 *t*(15) = 6.42, *P *<* *.001	.63 ± .02 *t*(15) = 5.45, *P* < .001
BA 1/2/3ab	.76 ± .02 *t*(15) = 1.27, *P *<* *.0001	.73 ± .03 *t *=* *7.76, *P *<* *.001
BA 4p	.73 ± .03 *t *=* *7.76, *P *<* *.001	.67 ± .03 *t*(15) = 6.004, *P *<* *.001
BA 6	.79 ± .03 *t*(15) = 11.55, *P *<* *.001	.75 ± .03 *t*(15) = 2.07, *P *<* *.001
BA 44/45	.68 ± .03 *t*(15) = 7.09, *P *<* *.001	.66 ± .03 *t*(15) = 5.86, *P < *.001
Control ROI	.52 ± .03, *t *=* *.77	

SVM, support vector machine; ROI, regions of interest; SPOC, superior parieto‐occipital cortex; SPLap, superior parietal lobe; BA, Brodmann area; WHG, whole hand grasping; hAIP, anterior part of the human intraparietal sulcus.

The classifier analyses showed that, independently form the object size, it was possible to linearly discriminate between the WHG and the Reaching‐only conditions from the voxel pattern activity of all the selected ROIs with the exception of the control ROI (see Table 3; Fig. 2, panel D).

For investigating possible interhemispheric asymmetries for the selected ROIs, we performed an RM‐ANOVA on the classifier accuracy using ROI (SPOC, SPLap, hAIP, BA 1/2/3ab, BA 44/45, BA 4p, and BA 6) and hemisphere (left vs. right) as within‐subject factors. The analysis revealed a main effect of ROI (*F*(6, 90) = 14.66, *P* < 0.001, $\eta_{p}^{2}$ = 0.49) and hemisphere (*F*(1, 15) = 10.96, *P *=* *0.005, $\eta_{p}^{2}$ = 0.42). The two‐way interaction was not significant (*F *=* *0.82). Independently from the selected ROI, classifier accuracy was higher when decoding from the left (*M* = 0.72 ± 0.01) than from the right (*M* = 0.68 ± 0.01) hemisphere. Paired *t*‐tests (FDR corrected, corrected *α* = 0.033) showed higher decoding accuracy from BA1/2/3ap (*M* = 0.79 ± 0.02 SEM) with respect to all of the other ROIs (all *t*s > 3.81, all *P*s < 0.002) except for the BA 6 (*M* = 0.77 ± 0.02 SEM, *t *=* *1.004). Moreover, also decoding from BA 6 was more accurate than from all of the other ROIs (all *t*s* *≥ 2.4, all *P*s ≤ 0.029). In contrast, decoding accuracy from SPOC (*M* = 0.6 ± 0.02 SEM) areas was lower than that from all of the other ROIs (all *P*s < 0.002) except for BA 44/45 (*M* = 0.67 ± 0.03 SEM, *t *=* *2.27). Moreover, decoding from BA 4p (*M* = 0.7 ± 0.02 SEM) was more accurate that from hAIP (*M* = 0.66 ± 0.02 SEM, *t*(15) = 2.52, *P *=* *0.023) (see Fig. 3, panel C). No further significant results were observed.

### Classification comparison

As a final step we compared the decoding accuracies among the three possible classifications (i.e., Grasp type, PG vs. Reaching‐only, and WHG vs. Reaching‐only). We computed a RM‐ANOVA on the classifier accuracy using Classification (three levels) as within‐subject factor, separately for each ROI (SPOC, SPLap, hAIP, BA 1/2/3ab, BA 44/45, BA 4p, and BA 6). The analysis revealed for all the ROIs, except for SPOC areas (*F *=* *2.44, *P *=* *0.1), a main effect of Classification (all *F*s ≥ 6.58, all *P*s < 0.004, all $\eta_{p}^{2}$ ≥ 0.31). A significant linear contrast (all *F*s ≥ 13.14, all *P*s < 0.002, all $\eta_{p}^{2}$ ≥ 0.47) for all the ROIs, suggests that the decoding accuracies linearly increased from the Grasp type toward PG versus Reaching‐only and WGH versus Reaching‐only classifications.

## Discussion

Here, we exploited the potential of MVPA for better characterizing the specific contribution of brain areas belonging to the reaching–grasping network in humans. We performed MVPA on the activation patterns detected within ROIs of a wide frontoparietal network, for investigating three main aspects characterizing reaching‐only and reach‐to‐grasp actions: the role of object size, the grasp type, and the congruence between the grasp type and the object size. In addition, to better define a possible differential contribution of grasping‐related areas, we also directly compared reach‐to‐grasp and reaching‐only actions.

Results showed no critical role of object size in performing both reach‐to‐grasp and reaching‐only actions. It was possible, however, to discriminate between grasp types (PG and WHG) regardless of the object size from activation patterns within all the selected ROIs, with the exception of bilateral SPOC and right hAIP. No effects were found concerning the congruence between grasp type and object size. Distinctions between reach‐to‐grasp (PG and WHG separately) and reaching‐only actions emerged from all the selected ROIs. Overall, decoding accuracy was higher in distinguishing reach‐to‐grasp from reaching‐only than in distinguishing PG from WHG actions. In both cases the left (controlateral) hemisphere played a prominent role in terms of decoding accuracy.

### Object size in reach‐to‐grasp action

The evidence that object size did not play a relevant role in reach‐to‐grasp action is consistent with the findings of the reference study by Begliomini et al. (2007b), where the GLM did not reveal a modulation of the BOLD activity induced by object size. This allows us to discard the hypothesis that object size may account for the differential activations within key areas concerned with visuomotor reach‐to‐grasp actions. This is, however, in contrast with a very recent finding of Monaco et al. (2015), where the authors used fMRI adaptation for investigating whether object size and location play a significant role in reach‐to‐grasp actions. Specifically, left hAIP showed adaptation effect only to object size, whereas left SPOC, primary somatosensory and motor areas (S1/M1), PMd and SMA were sensitive to both object size and location. This discrepancy could be ascribed to several factors. First, the paradigm of Monaco and colleagues was specifically conceived to highlight adaptation phenomena. Indeed, the systematic variation intrinsic properties (e.g., object size) of the stimulus is crucial for adaptation mechanisms. In contrast, in the study of Begliomini et al. (2007b) this aspect was manipulated in a different way (i.e., object size was kept constant within each run). Crucially, participants were informed about the size of the object to be grasped at the beginning of each run (i.e., small object in the odd runs, and large object in the even ones).

### Object size in reaching‐only action

The fact that no critical role of object size emerged for the reaching‐only action is in contrast with recent findings by Tarantino et al. (2014). The authors registered kinematic and evoked related potentials while participants were asked to reach‐only for differently sized objects. Results showed that the kinematics of reaching‐only action, as well as the amplitude and the latency of P300 and N400 ERP components in parietal and prefrontal sites, respectively, were modulated by object size, consistent with physiological findings on nonhuman primates (Fattori et al. 2012). The discrepancy between these and our findings could rely on the better temporal resolution provided by ERPs with respect to fMRI, and might suggest that object size, or more precisely the level of accuracy of the movement determined by it, could modulate reaching‐only actions on a temporal, rather than a spatial basis.

### Grasp type

Concerning the grasp type, here we showed that discrimination between PG and WHG is possible from several areas of our selected network. In particular, decoding accuracy was higher within the left (contralateral) rather than the right (ipsilateral) hemisphere. Conventional univariate analyses performed by Begliomini et al. (2007b) revealed only an effect of grasp type (i.e., [PGS + PGL] > [WHGS + WHGL]) in the left hAIP. This discrepancy could be ascribed to the differences between univariate and multivariate analysis and to the fact that the findings by Begliomini et al. (2007b) were obtained by means of a subtraction procedure (reach‐to‐grasp—reaching‐only) which is conventionally adopted by studies focusing on visuomotor transformation components underlying grasping (Culham et al. 2003, 2006). Here we confirmed the involvement of left AIP in coding differences between the two types of grasp, also at the level of voxel patterns. Moreover, MVPA revealed that other ROIs were involved in grasp type coding (all but bilateral SPOC and right hAIP), because activity modulation within the voxel patterns related to the two conditions were linearly separable within each ROI.

Evidence that neurons within hAIP can selectively code for different grasp types comes from neurophysiological studies (Murata et al. 2000). Although there is evidence for different levels of activity depending on type of grasp within the human hAIP (Begliomini et al. 2007a,b), whether the human hAIP contains neural populations selectively involved in the coding of different grasping schemata remained to be clarified. Here we demonstrate that only left hAIP can discriminate between grasp types. This result is in agreement with the study by Gallivan et al. (2011), the first using a decoding method for discriminating between different types of precision grasping (toward a small vs. a large object stacked in a top and bottom location, respectively). However, Gallivan et al. (2011) did not include the right hAIP in their decoding analysis, neglecting a possible role of the ipsilateral hemisphere in coding for different grasp types. Their ROI selection procedure was relying on the results of the GLM group random effects voxelwise analysis. Despite the fact that they avoided the “double dipping” problem (Kriegeskorte et al. 2009) by performing this analysis on a different data set that was not used for decoding analysis, this ROI selection procedure suffers from the limitations of the GLM and it implies discarding all regions that do not show significant effects at the level of single voxel analysis. However, in our study, MVPA showed that no grasp type discrimination is possible from right hAIP.

Critically, we did not find any involvement of SPOC areas in distinguishing PG from WHG. Evidence of the involvement of left SPOC in discriminating between two different precision grasping comes from the study of Gallivan et al. (2011). Because in that study also object position was manipulated, it is unclear whether this result is due either to the object size or to a different direction in reaching toward the bottom or top cube. The spatial aspect is crucial since it has been demonstrated that SPOC activity is strictly related to the transport component of the reach‐to‐grasp action (Cavina‐Pratesi et al. 2010). In contrast, our results are consistent with the study of Fabbri et al. (2014), in which the comparison between PG and WHG actions toward a spherical object of constant size did not reveal any grasp type selectivity for the left SPOC. However, Fabbri et al. (2014) focused their attention only on the left hemisphere, discarding possible results within the right hemisphere, whereas here we show that the lack of grasp type selectivity characterizes both contralateral and ipsilateral SPOC.

The contribution of the SPLap in discriminating precision versus whole hand grasp actions is consistent with the findings of Fabbri et al. (2014). Specifically, SPLa broadly corresponds to monkey ventral intraparietal area (VIP; Mars et al. 2011) and has been reported to be sensitive to the spatial congruency between visual and tactile information (Duhamel et al. 1998).

The involvement of bilateral BA 1/2/3ab in coding the grasp type could be explained by the sensitivity to different somatosensory feedback provided by the two grasping actions (i.e., PG and WHG). This peculiarity is confirmed by the results obtained for the bilateral dorsal premotor (BA 6) and motor (BA 4p) cortices: somatosensory information from the hand should be integrated with motor commands from frontal motor areas specifying the type of movement necessary to achieve the goal of grasping (Gardner et al. 2007).

On the basis of neurophysiological and neuroimaging studies, the role of the PMd for distal forelimb movements is becoming increasingly established (Raos et al. 2004; Begliomini et al. 2007b). Here we extend this literature by demonstrating that within the left BA 6 different patterns of activity associated to different grasp types are evident. This is in agreement with neurophysiological findings showing that F2 and F5 share similar functional properties and act in concert for the control of grasping (Raos et al. 2004, 2006). In particular, F5 would be mainly devoted to grasp selection, while F2 would monitor hand shaping during the ongoing movement, assuring movement accuracy. Therefore, it might well be that the discrimination ability shown here by left BA 6 indicates a differential hand shape monitoring depending on grasp types. Grasp type classification was also possible within the right BA 6: as demonstrated by previous findings, this result could be explained in terms of learning new motor sequences or by high requirements in terms of precision and coordination, independently from the hand used (Davare et al. 2006; Begliomini et al. 2008). In this regard, PG requires high precision in positioning the two fingers on the opposite sides of the object, whereas WHG requires coordination among phalanxes of all fingers. Therefore, it is conceivable that the right BA 6 acts in concert with the left BA 6, in order to fulfill the accuracy and coordination requirements intrinsic to the considered types of grasp (Begliomini et al. 2007b).

Neurophysiological data suggest a key role for PMv in selecting the most appropriate motor configuration on the basis of 3D analysis provided by AIP (Fagg and Arbib 1998). In this respect, human neuroimaging findings have provided mixed results. Whereas isometric grasping tasks detected PMv activity (Ehrsson et al. 2001), visually guided tasks did not (Culham et al. 2006; Begliomini et al. 2007a,b). Therefore, it was unclear whether the human PMv really holds a function of “motor vocabulary” similarly to macaque F5. Our results extend this literature by showing that in humans bilateral BA44/45 exhibits a differential activation pattern in association with different grasp types and supports the parallelism between macaque and humans in grasp type selectivity at the level of premotor cortices (Murata et al. 1997; Carpaneto et al. 2011). Furthermore, several functional imaging studies have shown activation in both the left and right PMv when subjects manipulated (Binkofski et al. 1999) or grasped (Ehrsson et al. 2000, 2001) objects. Bilateral involvement of PMv during grasping movements has been also observed in TMS studies (Davare et al. 2006, 2008) revealing that lesioning either the left or the right PMv modifies fingertip positioning, which is a prerequisite to grasp an object properly (Sartori et al. 2011).

Previous neurophysiological data report grasp type specificity within M1. M1 neurons active during WHG are silent during PG (e.g., Muir and Lemon 1983). Although in humans different levels of activity in M1 for PG and WHG (Ehrsson et al. 2001; Begliomini et al. 2007a) have been reported, different spatial distributions of activity associated with different grasping schemata had yet to be demonstrated. Here we showed that the bilateral BA 4p significantly discriminated among grasping schemata. Since the movement is performed with the right hand, one might have expected this functional property to be evident solely in left BA 4p. In general, however, the contribution of the ipsilateral hemisphere could be hidden when using traditional GLM analysis, since in this case the research question is based on searching where in the brain there is a significant greater BOLD activity for an experimental condition with respect to a second one. This assumption could discard the involvement of brain areas where the experimental manipulations produce an effect at the level of activation patterns rather than at the level of single voxel activity. In contrast, MVPA is intended to uncover whether and to what extent a brain area is coding differential voxel pattern representations for two experimental conditions. We found that also the ipsilateral hemisphere has a role in representing different grasp types, but the decoding accuracy was significantly higher in left than in the right BA4p. Recent findings show that administering rTMS (repetitive TMS) on ipsilateral M1 affects the timing of muscle recruitment, resulting in a loss of coordination during hand movement (Davare et al. 2007). This phenomenon potentially occurs on the basis of reciprocal connections between cortices via the corpus callosum (Boroojerdi et al. 1996; Di Lazzaro et al. 1999).

Overall, we showed, for the first time, that grasp type could be decoded from a wide frontoparietal network in both hemispheres, with the left (controlateral) hemisphere playing a more informative role with respect to the right (ipsilateral) one. However, since participants were able to see their own movements, results about grasp type could be also interpreted as different representations mediated by the vision of a different movement.

### Congruence

Despite the fact that in the study by Begliomini et al. (2007b) the contrast between natural and constrained reach‐to‐grasp actions (i.e., our Congruence classification) revealed a greater activation within few voxels belonging to bilateral PMd and left M1, this was not the case with MVPA. Thus, a difference between congruent (i.e., PGS and WGHL) and incongruent (PGL and WHGS) grasping actions could be revealed only in terms of univariate analysis, whereas no activation pattern within wider brain areas encoded this difference. This might stem from a limit of MVPA in distinguishing patterns of activity across a large set of voxels (i.e., large‐size ROIs) when the discriminating information is encoded in a small percentage of the input voxels. MVPA is more sensitive to distributed coding of information whereas univariate analysis is more sensitive to global engagement in ongoing tasks (Jimura and Poldrack 2012). Another possible explanation for the lack of the congruence effect, could rely on the fact that we did not apply spatial smoothing to fMRI data before MVPA. As recently shown in a study based on simulated data (Stelzer et al. 2014), the combined use of spatial smoothing and cluster based correction could increase the number of false positives and false negatives, respectively. Thus, both univariate and multivariate approaches could introduce possible limitations, and their combination should be more informative than the use of a single approach (see also Gallivan et al. 2011 for a similar argument).

### Reach‐to‐grasp versus reaching‐only actions

Here, we showed that it was possible to discriminate between reach‐to‐grasp and reaching‐only actions from the selected frontoparietal network. Interestingly, a prominent role in characterizing the reaching–grasping network is played by bilateral SPOC and right hAIP. These areas were not sensitive in decoding grasp type, but played a significant role in discriminating between reach‐to‐grasp and reaching‐only actions.

Our results on SPOC suggest that the contribution of these areas might be more crucial for reaching‐only than shaping the fingers for different grip types, which is consistent with the findings of Cavina‐Pratesi et al. (2010). These authors reported that the human SPOC showed stronger activation during reach‐to‐grasp action toward far rather than near locations, suggesting a preference for the transport rather than the grasp component. However, our results are in contrast with those reported by Fabbri et al. (2014), where left SPOC did not show any effect in discriminating between reach‐to‐grasp and reaching‐only actions.

Our results on right hAIP suggest that this area contributes to the representation of both reaching‐only and reach‐to‐grasp actions, but it does not appear to be critically involved in the finer distinctions between grasp types. This latter result might indicate a major role of the right hAIP in visuomotor reaching‐only rather than grasping action representation.

The contribution of SPLap in reaching‐only and reach‐to‐grasp actions was not surprising. This result is consistent with those of Fabbri et al. (2014) and it can be explained by the fact that this area is sensitive to the direction of visual, tactile and auditory stimuli (Bremmer et al., 2001). Indeed, in our experiment, participants were informed on the type of action to be performed (e.g., reach‐to‐grasp vs. reaching‐only) by auditory cues.

The fact that BA1/2/3ab, bilaterally, was involved in the discrimination between reaching‐only and reach‐to‐grasp actions could be explained by a sensitivity to different somatosensory feedback provided by the two actions toward the object. In addition, this was indexed by higher accuracy in discriminating between reaching‐only and reach‐to‐grasp using WHG rather than PG, probably mirroring a greater difference in hand configuration, and hence in somatosensory feedback.

The involvement of BA44/45 in discriminating between reaching‐only and reach‐to‐grasp actions is consistent with the most recent findings of Fabbri et al. (2014) and Gallivan et al. (2011), both using multivariate approaches for analyzing fMRI data. The first study highlighted that reach direction and grip type are both represented in left PMv, whereas the second one showed that left PMv was involved in the discrimination between precision grasping and touching, in both the planning and the execution phase of the actions.

The contribution of bilateral BA 6 in distinguishing between reach‐to‐grasp and reaching‐only actions is not surprising, since this area has been firstly suggested to code only for the transport phase of the hand toward an object (i.e., reaching) (Begliomini et al. 2014; Culham et al. 2006; Vesia and Crawford, 2012) and has been shown to be involved in the representation of both the transport and the hand preshaping components of reaching‐only and reach‐to‐grasp actions, respectively (e.g., Fabbri et al. 2014). The bilateral involvement of PMd in coding direction and amplitude of reaching‐only has been shown by Fabbri et al. (2012), thus it was not surprising that different activity patterns are present in these areas for reaching‐only and reach‐to‐grasp actions.

Finally, the involvement of primary motor area in reach‐to‐grasp versus reaching‐only discrimination was expected, as well as its involvement in distinguishing finer aspects of the grasping action (i.e., grasp type classification). These results are consistent with the most recent neuroimaging studies in humans (Gallivan et al. 2011; Fabbri et al. 2012, 2014). Interestingly, the novelty of these results relies on the right (ipsilateral) contribution of BA4p. As in the case of the grasp type classification, we found a bilateral involvement of BA4p in discriminating between reaching‐only and reach‐to‐grasp actions, even if the left (contralateral) hemisphere played a prominent role, in terms of classification accuracy.

In conclusion, our results showed significant hemispheric asymmetries in discriminating reaching‐only from reach‐to‐grasp actions and PG from WHG, which consisted of a left (i.e., contralateral) hemisphere dominance. This is consistent to our expectations, since participants were using the right hand to perform the actions. Furthermore, we found that somatosensory and dorsal premotor areas were more responsive in distinguishing between reaching‐only and reach‐to‐grasp actions, with respect to all the other areas within the selected network. Finally, within the selected network, decoding accuracy was higher when discriminating reaching‐only from reach‐to‐grasp action, when using WHG rather than PG. This result, together with the fact that no critical role was played by object size, could suggest that different activation patterns underlying reach‐to‐grasp and reaching‐only actions could be mainly due to a physical difference in hand configuration. The fact that this information was probably guiding the discrimination within all the selected network (including parietal areas) indicates that hand preshaping begins in early stages of action planning (i.e., action preparation), as also suggested by Gallivan et al. (2011) and Begliomini et al. (2014).

## Conclusion

To summarize, in our study no critical role of object size emerged for both reaching‐only and reach‐to‐grasp actions. This result runs against the hypothesis that the intrinsic object properties (e.g., object size) could play a key role in both reach‐to‐grasp and reaching‐only actions. Here we showed, for the first time that grasp type (i.e., PG vs. WHG), independently from object size, can be reliably discriminated by a linear classifier within a wide frontoparietal network distributed across both the hemispheres, with the exception of SPOC areas and right hAIP. The left (i.e., controlateral) hemisphere, however, played a crucial role in terms of decoding accuracy. No significant interaction between the grasp type and the object size (i.e., our congruence classification) emerged within the considered network, despite the fact that univariate analysis of the same data set (Begliomini et al. 2007b) showed that activity of few voxels within PMd and M1 areas was modulated by congruence. This highlights the importance to perform data analysis from a more comprehensive perspective, combining both univariate and multivariate analyses. This integrated approach could provide more informative results and a deeper understanding of the neural dynamics underlying the cognitive processes of interest. Finally, our results provided further evidence against the hypothesis of a clear‐cut distinction between a dorsomedial (e.g., SPOC, medial intraparietal area MIP, and PMd) and dorsolateral (e.g., hAIP and PMv) pathways, specialized for reaching‐only and reach‐to‐grasp actions, respectively, as reported in a series of recent studies on human and nonhuman primates (Fattori et al. 2009, 2010; Cavina‐Pratesi et al. 2010; Monaco et al. 2011). Our results are also consistent with the findings of Grol et al. (2007), which argue against the presence of dedicated cerebral circuits for reaching‐only and reach‐to grasp actions, suggesting that the contributions of the dorsolateral and the dorsomedial circuits are a function of the degree of online control required by the movement. Finally, our results are perfectly consistent with the theory of a dorsomedial visual stream involved in reach‐to‐grasp actions, suggested by Galletti et al. (2003) in nonhuman primates, and well documented by Fattori et al. (2009, 2010). Reaching‐only and reach‐to‐grasp actions could be better characterized by temporal, rather than spatial criteria across planning and execution stages of the action, as also suggested by a recent study of Begliomini et al. (2014). Here we showed that several areas of the human reaching–grasping network are involved in processing aspects related to both reach‐to‐grasp and reaching‐only actions. Crucially, the precise nature, in terms of timing and direction (causality—Davare et al. 2010; Grol et al. 2007) of the relations between the involved brain areas remains to be clarified by future studies.

Altogether, the findings provided by the integrated approach adopted in this work enrich the current knowledge regarding the functional role of key brain areas involved in the cortical control of reaching‐only and reach‐to‐grasp actions in humans, by revealing novel fine‐grained distinctions among action types within a wide frontoparietal network.

## Conflict of Interest

None declared.
